# Control of Porous Layer Thickness in Thermophoretic Deposition of Nanoparticles

**DOI:** 10.3390/ma14092395

**Published:** 2021-05-04

**Authors:** Malte Schalk, Suman Pokhrel, Marco Schowalter, Andreas Rosenauer, Lutz Mädler

**Affiliations:** 1 Faculty of Production Engineering, University of Bremen, 28359 Bremen, Germany; m.schalk@iwt.uni-bremen.de (M.S.); spokhrel@iwt.uni-bremen.de (S.P.); 2 Leibniz Institute for Materials Engineering IWT, 28359 Bremen, Germany; 3 Institute of Solid State Physics, University of Bremen, 28359 Bremen, Germany; schowalter@ifp.uni-bremen.de (M.S.); rosenauer@ifp.uni-bremen.de (A.R.)

**Keywords:** flame spray pyrolysis, nanoparticles, thermophoretic deposition, film thickness

## Abstract

The film thickness plays an important role in the performance of materials applicable to different technologies including chemical sensors, catalysis and/or energy materials. The relationship between the surface and volume of the functional layers is key to high performance evaluations. Here we demonstrate the thermophoretic deposition of different thicknesses of the functional layers designed using flame combustion of tin 2-ethylhexanoate dissolved in xylene, and measurement of thickness by scanning electron microscopy and focused ion beam. The parameters such as spray fluid concentration (differing Sn^2+^ content), substrate-nozzle distance and time of the spray were considered to investigate the layer growth. The results showed ≈ 23, 124 and 161 μm thickness of the SnO_2_ layer after flame spray of 0.1, 0.5 M and 1.0 M tin 2-EHA-Xylene solutions for 1200 s. While Sn^2+^ concentration was 0.5 M for all the flame sprays, the substrates placed at 250, 220 and 200 mm from the flame nozzle had layer thicknesses of 113, 116 and 132 µm, respectively. Spray time dependent thickness growth showed a linear increase from 8.5 to 152.1 µm when the substrates were flame sprayed for 30 s to 1200 s using 0.5 M tin 2-EHA-Xylene solutions. Changing the dispersion oxygen flow (3–7 L/min) had almost no effect on layer thickness. Layers fabricated were compared to a model found in literature, which seems to describe the thickness well in the domain of varied parameters. It turned out that primary particle size deposited on the substrate can be tuned without altering the layer thickness and with little effect on porosity. Applications depending on porosity, such as catalysis or gas sensing, can benefit from tuning the layer thickness and primary particle size.

## 1. Introduction

Wet chemical routes (drop coating, dip and/or spin-coating, and screen printing), vapor deposition technique (chemical vapor deposition, physical vapor deposition, plasma deposition), and direct deposition of the nanoparticle aerosol stream (e.g., flame spray pyrolysis, spray pyrolysis) are major technologies for thin and thick film coating [1,2,3,4,5,6,7]. Every technique is specific to the nature of the layer structure, thickness and mechanical stability [8]. The screen-printed layers using homogeneous pastes (mixture of the active material, organic binders and/or solvents) are sintered at required temperatures to evaporate the organic components used for making the paste [9]. The capillary and surface forces associated with vaporization of the highly volatile components from the surface layer induce substrate bending (when the substrates are flexible) and/or surface cracks on the fabricated layers [10]. Thus, fabrication of crack-free layers with application specific thickness is difficult using traditional approaches [11]. Furthermore, the porosity of, for example, screen printed layers, depends on initial particle size [12]. Chemical vapor deposition (CVD) for printing layers has the following limitations and challenges: (1) long processing time—in the range of hours; (2) requirement of high temperature—in the range of 450–1200 °C, depending on the substrate material; (3) possibility of the material reacting with the substrates; (4) limitation using high temperature stable substrates [13]. Control over layer properties including porosity and primary particle sizes is also difficult. In the flame aerosol technology, thermophoretic deposition is the dominant layer formation mechanism and the deposition rate is directly controlled by the temperature gradient of the aerosol stream and the substrate [11,14]. Such thermophoretic deposition offers several advantages in comparison to wet chemistry processes or vapor deposition techniques including: (1) possibility of a single step gas phase deposition avoiding any post-treatment such as evaporation or drying of liquid components used; (2) self-forming aggregates during the gas phase deposition leading to crack-free layers; (3) overall short processing times (especially compared to CVD). The layer thickness is achieved by selection of correct precursor-solvent combination with high enthalpy density of the spray solution [15], concentration of the metal precursor, the deposition time, the nozzle-to-substrate distance and the amount of dispersion oxygen. Although thermophoretic deposition is an attractive technique for large-scale coatings, the loose particle-substrate contact (low mechanical stability with higher porosity in comparison to screen printed layers) and the difficulty in coating temperature sensitive substrates are major drawbacks. To improve mechanical stability of the thermophoretically deposited layers, a process capable of transferring porous layers to various substrates using a pressure based role-to-role lamination technique at room temperature was developed [16]. Such a fabrication process allowed thermophoretically deposited layer transfer from the particle collecting unit even to the flexible substrates [17,18].

The mass transfer rate and/or electron diffusion pathways within the layers are directly related to the film porosity [19,20,21]. The highly porous nanoparticle layer (ratio of empty volume of the substrate to total volume after spray) with specific thickness is applicable to gas sensing [22,23,24,25,26,27]. Based on the literature, the best chemical sensing performance is realized using nanoparticle layer thickness of 10–40 μm [28]. However, achieving the flame parameters for creation of tailor-made layers for various applications is still in its premature stage and requires standardization of the flame parameters and spray settings.

While only few reports in the literature describe the actual thickness necessary for the sensing performance (10–40 μm by Mädler et al. and 30 μm by Kemmler et al.) [28,29], there is a pressing need to establish a technique for in-situ fabrication of a nanoparticle layer with precise layer thickness and controlled porosity. Hence, in the present investigation the layers are fabricated via variation of: (1) metal concentration in the spray solution; (2) nozzle distance from the sensor substrates; (3) time of spray on the sensor substrates (4); and dispersion oxygen flow. In addition to previous work, we also examine porosity of our samples.

## 2. Experimental

### 2.1. Flame Spray Pyrolysis and Layer Fabrication

The SnO_2_ nanoparticles were prepared from 3.3 mL of tin 2-ethylhexhanoate (99.5% pure, Sigma-Aldrich (represented by Merck KGaA in Germany, Darmstadt, Germany) in 96.7 mL xylene solution. Four different experiments for the investigation of the layer thickness were performed: (1) Sn concentration vs. layer thickness; (2) nozzle height vs. layer thickness; (3) time vs. layer thickness; (4) dispersion oxygen vs. layer thickness.

For the parameter study Sn concentration vs. layer thickness, 3.3, 16.52 and 33.05 mL tin 2-ethylhexanoate were dissolved in 96.7, 83.48 and 66.95 mL of xylene, respectively, to obtain 0.1, 0.5 and 1.0 M Sn concentration. For the flame spray experiments using different nozzle height, (substrates placed at 200, 220 and 250 mm from the nozzle), 0.5 M Sn solutions was used. For determining the influence of spray time (30, 60, 300,600 and 1200 s) with respect to film thickness, 0.5 M Sn solution was used with a constant nozzle-substrate distance of 250 mm. All these precursor solutions were fed to the nozzle with a syringe pump (KD Scientific, KDS-100-CF) and combusted with premixed CH_4_ + O_2_ (1.5 L/min + 3.2 L/min) along with 5 L/min dispersant O_2_ gas for the first three experiments and 3, 4, 5, 6 and 7 L/min dispersant O_2_ gas for investigation of the dispersion gas at a constant pressure drop of 1.5 bar at the nozzle. The particles were formed by reaction, nucleation, surface growth, coagulation and coalescence in the spray flame environment and collected on a filter (Pall (represented by VWR Chemicals in Germany, Darmstadt, Germany), Type A/E, 257 mm diameter) placed 600 mm above the nozzle using a vacuum pump (Busch SV 1025 C 0000 IKZZ, Maulburg, Germany) [30,31]. For the thermophoretic deposition, the substrates were fixed on a brass support facing down towards the flame at the required height from the nozzle. The temperature of the thermophoretic deposition was monitored using a thermocouple in combination with a variable area flow meter (ABB) assuring a substrate temperature > 120 °C to avoid any water condensation on the fabricated layers.

### 2.2. BET, XRD and SEM-FIB Measurements

The powder X-ray diffraction (XRD) patterns were recorded for all the samples on a Bruker D8 Discover (Karlsruhe, Germany), equipped with a Cu-tube producing Ni-filtered K_α1,2_ radiation. The samples were prepared in ≈0.2 mm deep and ≈14 mm wide blind holes on single-crystalline Si holders. Diffraction patterns were taken from 5 to 135° 2*θ* and 1.5 s time steps without sample spin. The BET-surface adsorption measurements (to acquire specific surface areas) were performed using at liquid N_2_ temperature on a Quantachrome NOVA 4000e gas adsorption system (Quatachrome represented by Anton-Paar, Ostfildern-Scharnhausen, Germany) for acquiring specific surface areas. The measurement cells with ~70 mg of each powder were loaded in the degassing chamber and kept at 200 °C for 2 h. The data were collected by adsorbing/desorbing the known volume of the gas at pressure ranging from 0.01 to 0.90 and at the temperature of 77 K. The layer thickness was investigated using a Nova200 dual beam instrument from FEI (Hillsboro, OR, USA) and an Auriga cross beam from Zeiss (Oberkochen, Germany). The first attempt was to cut “cleaning cross sections” into the material using the Ga-column of the Nova200 and measure the thickness of the layer in side view under an angle of 52° using the electron beam. Later substrates were broken into parts and mounted in the Auriga SEM in such a way that the surface normal of the sample could be aligned almost perpendicular to the electron beam. The acceleration voltage was chosen to be 5 kV. Previous work focused on determination of the deposited mass only. By measuring the layer height simultaneously with weight and cross sectional area of the layers, it is possible to calculate the porosity of the thermophoretically deposited layer.

## 3. Results and Discussion

### 3.1. Particle Characterization

The BET surface area is related to the average equivalent primary particle size as [30]: *d*_BET_ = 6/(*ρ_p_*·*S_A_*), where *d*_BET_ is the average diameter of a spherical particle, *S_A_* represents the measured surface area of the powder in m^2^/g, and *ρ_p_* is the theoretical density in kg/m^3^. The specific surface area of SnO_2_ prepared using tin-2-ethylhexanoate-xylene solution with different concentrations including 0.1, 0.5 and 1.0 M (by Sn^2+^) is in the range from *S_A_* (*d*_BET_) = 108.9 (7.8) to 71.8 (11.9) m^2^/g (nm). The primary particle size increases from 7.1 nm to 11.9 nm with a ten-time increase in the concentration (from 0.1 to 1.0 M). When the concentration of spray solution was kept constant at 0.5 M and only the nozzle-substrate-distance (HAB) was varied, the specific surface area (88.8 (9.7 nm) m^2^/g to 83.2 (10.4 nm) m^2^/g) remained almost the same, as expected. The distance of the substrate and the layer growth seem independent from each other, although the substrate placed above the nozzle deviates some of the hot aerosol at the respective distances (HAB) before it reaches the collecting unit. In the third case, when the time of the spray was varied from 30 s to 1200 s, keeping the Sn^2+^ concentration constant at 0.5 M, the particle size varies insignificantly (only between 91.7 (9.2 nm) m^2^/g to 84.7 (10.2 nm) m^2^/g). When the dispersion oxygen was varied from 3 to 7 L/min, the particle size decreased from 11.9 nm to 6.3 nm. The lower dispersion oxygen flow results in longer flame height, with longer particle residence time in the hot region of the flame triggering larger particle size [32,33]. The primary particle sizes obtained from the BET measurements reasonably agree with the crystallite sizes obtained from Rietveld analysis of the XRD patterns (see Table 1).

**Figure 1:**
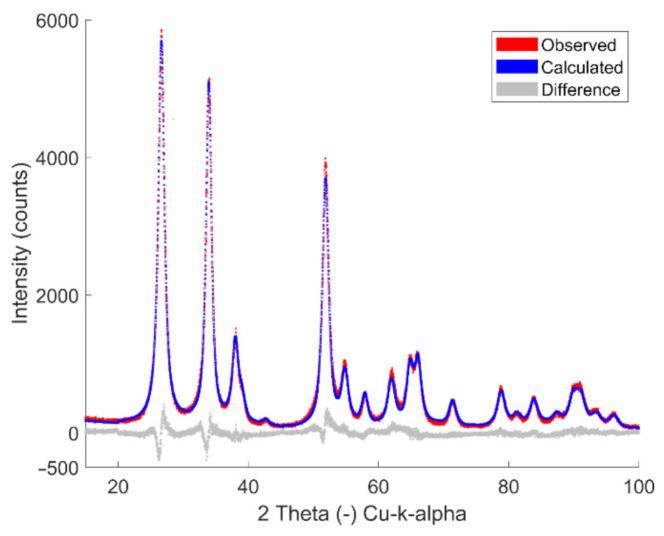
XRD data and Rietveld refinement (exemplary presentation) of SnO_2_ particles synthesized with 0.5 M and a deposition time of 1200 s with 200 mm distance and 5 L/min dispersion oxygen. The XRD refinement patterns of particles obtained at different flame parameters are presented in supplementary information.

The crystallite size determination of SnO_2_ nanoparticles (for different concentrations, different spray time, different nozzle heights and at different dispersed O_2_) were obtained with Rietveld refinement of the XRD patterns using SnO_2_ database entry code (ICSD 9163). The full profile fitting method was employed using BRASS program to obtain cell and microstructural parameters [34,35,36]. A typical Rietveld refined powder XRD pattern of an SnO_2_ sample is presented in Figure 1, and the XRD patterns of all the other SnO_2_ samples are shown in supplementary information (Figures S1–S4). From the full profile refinement, the overall characteristics of the powder patterns clearly agreed with ICSD 9163. In contrast, the apparent crystallite size depends on precursor concentration, flame nozzle-substrate distances and dispersion oxygen flow. From the refinement, the lattice constants are found to be close to those reported for SnO_2_, with reasonable agreement between the refined crystallite sizes (*d*_TEM_) and primary particle sizes (*d*_BET_). Li et al. investigated SnO_2_ particles obtained using the tin-2-ethylhexanote-xylene solutions with the same Sn^2+^ concentrations of 0.1, 0.5 and 1.0 M. The data showed high crystallinity of the particles in sizes ranging from 10 to 20 nm consistent with primary particle sizes (*d*_BET_) and crystallite sizes (*d*_XRD_) in this work [32].

The primary particle size (*d_p_*) is related to the dispersed oxygen flow during flame spray with volumetric particle concentration $C_{v}$ and residence time t i.e., dp≈Cvt25 [37]. The particle concentration is lower for higher dispersion oxygen flow. Additionally, since per unit of time more gas has to pass a defined volume of the spray cone, the residence time is also lower. Both effects combined lead to smaller primary particle sizes for higher dispersion oxygen flow. Consistently lower precursor concentration also leads to smaller particles. Variation of spray time does not affect particle size since neither $C_{v}$ nor t are affected. Changing the nozzle to substrate distance has a minor effect since due to the high velocity in relation to the distance varied, the influence on residence time is negligible. While volume concentration and the time are changing with the dispersed oxygen flow and the precursor concentration, the increase in the precursor concentration by 5 times would mean a 2 times increase in the primary particle size. Assuming primary particle size determined by XRD for 0.5 M as a reference value (8.9 nm), the size for 0.1 M is 60% smaller (3.6 nm) while for 1.0 M the increase is 24% (11.0 nm). From the correlation stated above, a theoretical decrease of 50% for 0.1 M and an increase of 38% for 1.0 M would be expected. The correlation dp≈Cvt25 requires estimation of the number concentration of primary particles, which is nonlinear related to precursor concentration [38].

### 3.2. Film Characterization

**Figure 2:**
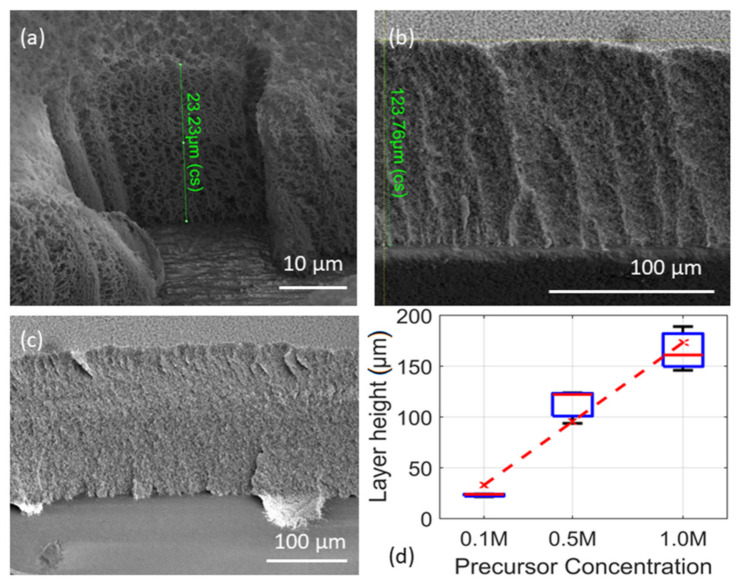
Thickness of layers (using SEM-FIB technique) obtained via gas phase combustion of different concentrations of (**a**) 0.1 M (**b**) 0.5 M, (**c**) 1.0 M of Tin 2-EHA-Xylene solutions followed by thermophoretic deposition. The SEM images show a focus ion beam cut-portion of the SnO_2_ layers (**d**) boxplot of the film thickness at different precursor concentration, red line is a linear fit with R^2^ = 0.95.

FIB is used to determine the (1) thickness of highly porous nanoparticle films, (2) cutting layers and (3) depositing materials for microelectromechanical systems (MEMS applicable in semiconductor industries) [39]. In this work the thickness of highly porous thermophoretically deposited layers were determined using such a technique. The film thickness obtained via flame combustion of 0.1, 0.5 M and 1.0 M tin 2-EHA-Xylene solutions were ~23, 124 and 161 μm, respectively, at the deposition time of 1200 s (see Figure 2a–c). Considering the film thickness model reported by Mädler et al., a higher concentration of particles in the gas phase should result in higher particle deposition flux and thus for equal spray time giving rise to thicker films.

This implication is verified with the thicker layer after 1200 s of spray for higher tin 2-EHA-Xylene concentration at otherwise unchanged flame conditions including substrate-nozzle distance and precursor flow [11]. The same holds true for 0.5 M concentration, indicating a systematic dependency. The primary particle size increases with higher 2-EHA-Xylene concentration and the larger particles tend to form layers with higher porosity [31].

**Figure 3:**
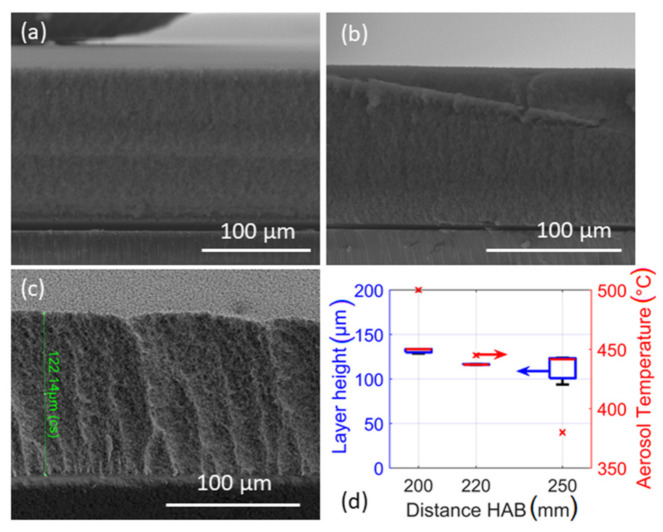
SEM images and results of experiments concerning distance as variable. Images show cross sections of layers sprayed at various distances; (**a**) 200 mm, (**b**) 220 mm, (**c**) 250 mm, pictures were taken with FIB and (**d**) Boxplot of acquired layer heights and temperature of the gas phase ≈ 1 cm below the substrate holder.

**Figure 4:**
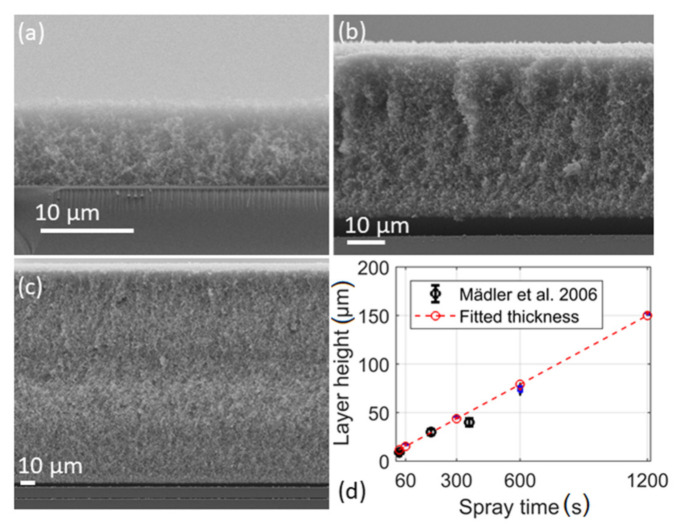
SEM images of the layers obtained at various spray times (**a**) 0.5 min, (**b**) 5 min, (**c**) 20 min with 0.5 M concentration of tin 2-ethyl hexanoate-xylene solution. (**d**) boxplot of the layer thickness acquired at different times of spray including data from Mädler et al. [11]. Red line was fitted with R^2^ = 0.997. Not shown are images of 1 and 10 min.

Other parameters such as the distance between the substrate and the nozzle might also influence the film thickness. To verify the hypothesis, the substrate was placed above the nozzle at different heights (200, 220 and 250 mm) for the film deposition, as can be seen from Figure 3a–d. For these three layer fabrication experiments, the concentration of the spray solution and deposition time were kept constant at 0.5 M and 20 min, respectively. While the mass and/or concentration of Sn^2+^ is the same for all the flame sprays, locating the substrate closer to the nozzle directly influences layer thickness, where Figure 4a–d illustrates the cross sections of the deposited particle layers at different nozzle heights. The mean layer height is larger when the substrate is placed closer to the nozzle. Considering the width of the boxes in the boxplot, the film thickness is obviously larger for substrates placed at a distance of 250 mm from the nozzle during flame spray. Additionally, in Figure 3d, right *y*-axis shows the change in the temperature of the substrate during the flame spray of the metal-free solvent at different substrate-nozzle distances. While a particle layer is formed on the tip of the thermocouple via precursor-solvent spray and a such layer on the temperature measuring probe significantly affects the real temperature, the usual approach is to measure the temperature with pure solvent [40]. Since the substrate holder was cooled to maintain the temperature at 120 °C (to avoid water condensation on the substrates), a higher temperature gradient of the particle aerosol stream enables greater thermophoretic force onto particles (resp. aggregates) and thus higher deposition flux [11]. In addition, the variation in layer height is also explained by the geometric effects of the spray. Since the total amount of metal is constant in all the spray solutions for all the sprays at different substrate-nozzle distances, the volumetric (particle) concentration has to decrease from nozzle (bottom) to filter (top) due to the conical aerosol stream. Hence, both effects enable a higher deposition rate when the substrate is closer to the nozzle. As expected, the crystallite sizes (*d*_XRD_) of the particles at different nozzle-substrate distances are very similar, ranging from 8.5 to 8.9 nm (see Table 1).

The spray and particle deposition times are directly related to obtained layer thickness. Considering spray times (0.5, 1, 5, 10 and 20 min) for the particle deposition, the thickness observed was 8.5, 16.8, 44.9, 74.3 to 152.1 µm, respectively. The SEM images of the cross sections of the layers deposited for 30, 300 and 1200 s show increased layer thickness with the deposition time (see Figure 4a–d). All layers are extremely uniform with only small deviations in height, as can be seen from the size of the boxes. In Figure 4b,c, it can be seen that the layer is detached from the substrate during sample probe preparation (via breakage) for cross sectional viewing in SEM.

**Figure 5:**
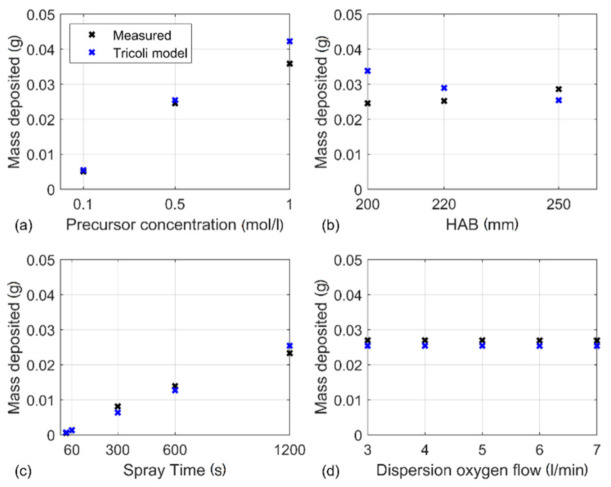
Comparison of the layer mass obtained by weighting the substrates prior to and post-deposition, and the model of Tricoli and Elmøe. (**a**) Comparison with regard to Precursor concentration and (**b**) comparison considering the height above burner, (**c**) comparison with regard to spray time and (**d**) comparison regarding dispersion oxygen flow.

Such detachment was not visible for the layers with lower thickness, e.g., layers obtained by flame spraying for 30, 60 and 300 s. In Figure 4d the plot shows the data acquired by Mädler et al. reporting nonlinearity of layer growth with deposition time. The data acquired in this work are best explained by a linear function where the linear fit resulted in a correlation coefficient of R^2^ = 0.997. Similarly, Tricoli and Elmøe came to the conclusion that the deposition rate is constant once the substrate temperature has reached a steady value [14]. According to their findings, the steady temperature is reached after 60 s, which is consistent with results from this work. However, the layer thickness for short spray times is more adequately described by the nonlinear model as shown by Mädler et al. Figure 5a shows deposited mass acquired by varying the precursor concentration and spray time, in comparison to a prediction based on the model of Tricoli and Elmøe [14]. The agreement between the experimental data and the value obtained from the model is good. For computation of layer mass, the respective variables such as HAB, entrainment air constant and equivalent nozzle diameter have been adapted to the FSP setup used.

The correct implementation of the model was verified using data from Tricoli and Elmøe and constants were given [14]. Regarding precursor concentration, the model seems to overestimate the deposited mass, especially for high concentration (see Figure 5a). For variation in spray time, the model seems to describe the mass deposited with a small nonsystematic error (see Figure 5c). The parameters’ nozzle to substrate distance (HAB) and dispersion oxygen flow can be described by the Tricoli and Elmøe model. When the distance between nozzle and substrate is varied the error between model and data acquired becomes significant (see Figure 5b).

**Figure 6:**
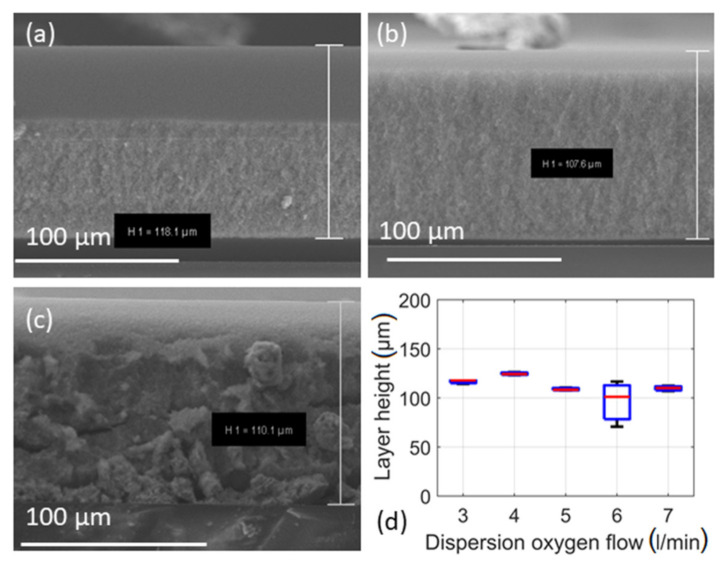
SEM images of the layers obtained at various dispersion oxygen flows. (**a**) 3 L/min, (**b**) 5 L/min and (**c**) 7 L/min. Additionally (**d**) shows the boxplot of the acquired data. Not shown are images of dispersion oxygen flows of 4 L/min and 6 L/min.

Figure 6d shows the layer thickness with respect to dispersion oxygen flow of 3, 4, 5, 6 and 7 L/min. The mean values in layer thickness differ only slightly from 116.7, 124.2, 108.8, 99.2 to 112.3 µm. The variation in layer thickness is about 20% while the flow had been more than doubled. However, Table 1 shows that primary particle diameter has decreased from 13.0, 12.6, 8.7, 8.9 to 8.4 µm. Throughout the experiments with variation in dispersion oxygen flow, the precursor concentration remained constant at 0.5 M. Due to the varied dispersion oxygen flow, the particle concentration in the lower parts of the spray cone is lower with higher dispersion oxygen flow. Assuming that 0.5 M and dispersion oxygen flow of 5 L/min is the reference value (9.0 nm), the parameters’ variations suggest an increase in particle diameter by 38% when lowering the dispersion oxygen flow to 3 L/min, and a decrease of 25% when increasing the dispersion oxygen flow to 7 L/min. The data from XRD in Table 1 show that the true increase was 32% (11.9 nm) and the true decrease 30% (6.3 nm).

**Figure 7:**
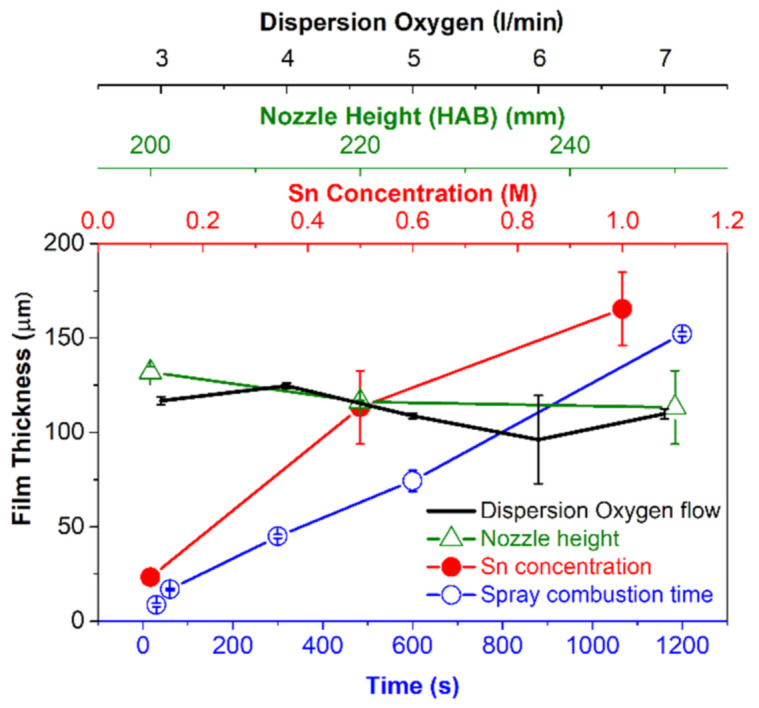
Comparison of the different layer thicknesses acquired. The zone (10 to 40 µm) which is relevant to gas sensor production has been highlighted. On the shared *y*-axis the layer thickness is displayed. Each parameter studied has an extra *x*-axis with the color corresponding to the respective graph. Error bars were computed by triplicate measurements of the respective layer.

It seems reasonable that the layer thickness is almost constant for the varied dispersion oxygen flow, because the total mass of metal in the spray is the same in this experiment. Variations in thickness could be attributed to the standard deviation commonly found in FSP experiments. Similar to the approaches made by Tricoli and Elmøe and Mädler et al., where linearity in layer thickness vs. time is a question of layer temperature [11,28], our data suggest a linear relationship as explained above (see Figure 4). In the model, the first data point at 30 s has been omitted due to unrealistic time conditions for layer fabrication. Even when including this data point, the correlation coefficient declines only slightly (R^2^ = 0.9969), suggesting a linear behavior for the entire spray times. The consideration of all the parameters studied in this work shows that the layer growth rate is specific to each parameter. However, the combination of parameters (precursor concentration, HAB, time, dispersion gas) has yet to be examined since the experiments conducted here were done for variation of one parameter only, i.e., the combination 200 mm HAB and 0.1 M is yet to be explored). Nevertheless, a set of parameters for standard precursor concentration 0.5 M of Sn^2+^ has been investigated. Although FSP is a potential synthetic tool for in-situ layer fabrication, limitations include the low precursor dissolution in solvents (for other metal oxides other than SnO_2_), and that precursor-solvent combinations limit the layer fabrication process [15]. Looking at the gradient of each line in Figure 7, one can identify if a change in the respective parameter has an influence on the layer thickness. A bigger gradient means that the layer thickness is more sensitive towards the respective parameter. For example, the layer thickness is more sensitive to changes in the precursor concentration than to changes in the distance. The error bars were computed by measurements of the respective layer in various locations and calculating the standard deviation. The smallest mean error is ±1.4 µm, found for variation in time, while for variation in concentration the mean error is the biggest with ±16.1 µm. The mean errors for layer height are ±6.25 µm for dispersion oxygen flow and ±6.75 µm for variation in nozzle height.

### 3.3. Porosity Determination

The porosity of all the layers obtained from different (1) spray solution concentration (2) substrate-nozzle distance (3) flame spray time and (4) dispersed O_2_ flow, were determined using ϕ = 1 − (m/(*ρ* *A* *h*)), where *ρ*, *A* and *h* are the density of SnO_2_ (obtained from the Rietveld analysis), area of the substrate coated and layer thickness, respectively.

**Figure 8:**
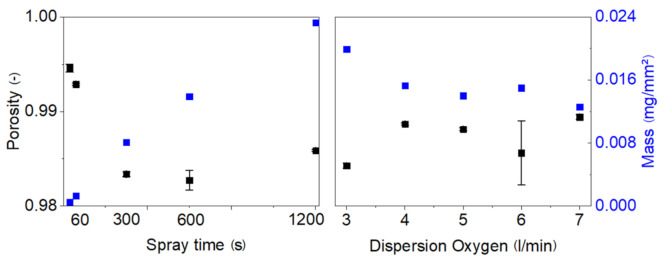
The mass of the particles deposited and the porosity of the layer after flame spray (**left**) Spray time vs. porosity/mass of the particles deposited (**right**) Dispersion oxygen vs. porosity/mass of the particle deposited.

Figure 8 left hand side shows the porosity and mass with respect to spray time. Initially, at short spray times, the porosity is higher than at 1200 s. At the beginning of an experiment in the absence of a particle layer on the substrate, the temperature of the substrate is equal to the temperature of the cooling block. During the spray duration a particle layer forms, which, due to its poor heat conductivity, is increasing in temperature. The thermophoretic force is therefore larger in the beginning of the experiment, leading to higher velocity of the aggregate respectively a higher Peclet Number. Consistent with findings from earlier work, the packing density of the deposited layer is higher with higher Pe. While mass of nanoparticles deposited on the substrate follows a linear trend similar to the layer thickness vs. spray time, the porosity is varying within 98–99.5% (see Figure 8, left panel). Looking at Figure 8 right panel, the porosity is also in the same range from 98.5 to 99.5%, although the mass of the particles deposited is varying.

The layers produced here are highly porous and in a thickness range of 100+ µm. The high porosity is beneficial for gas sensing applications when considering diffusion of target gases into the sensitive layer [41]. However, a drawback of these nanocrystalline structures is the fragility in terms of mechanical stability [6]. To overcome this problem is difficult, as any technique employed could potentially as well change the positive aspects, namely: primary particle size; the ability of target gas to interact with deep parts of the layer; and resistance of the acquired particle network by changing the bond co-ordination number [21]. Two approaches have been proposed to produce a mechanically stable layer that still has the desired gas sensing properties. One method developed was to laminate the layer directly from the filter to the substrate [16]. This technique allows for separation of the deposition and synthesis processes of nanoparticles, enabling the use of temperature sensitive substrates. It was found that the baseline resistance of sensors produced this way is significantly lower than for sensors produced by FSP direct thermophoretical deposition. The primary particle size had been conserved as well as the specific surface area of the particles. The effect of the lamination was to enlarge the number of particle-particle connections by decreasing the porosity from 89% to 80% [16,42]. Furthermore, it has been demonstrated that tuning the light absorption characteristics by lamination is possible [43].

Another approach was developed as an in-situ process for increasing the mechanical stability by annealing of the layers produced by conventional FSP [44]. After thermophoretic deposition the layer had been exposed to a particle free xylene flame for 30 s, exposing the layer to gas temperatures of approximately 1000 °C. This decreased porosity from 98% to 62% [44]. In contrast to the lamination process described above, the structure of the layers had been changed. A cauliflower-like structure had been observed. The sensor response was considered drastically improved. Furthermore, the response and recovery time had improved due to the improved transport of the test gas through the sensitive layer.

The method of annealing had later on been picked up and improved by switching from annealing after deposition to annealing during deposition, with modified feed rate and distance between substrate and nozzle. Temperatures of only slightly below 1200 °C have been reported [45]. Additionally, the resistance of the formed particle network had been monitored in-situ as proposed in [46]. The simultaneous deposition and sintering of the gas sensors had a lower baseline resistance compared to those annealed post-deposition. The response time was comparable for both annealing methods, while the overall sensor response was better for sensors annealed after deposition. All annealing techniques share the advantage of sintered particle necks. Another important factor is the contact of particles to the underlying substrate, where annealing during deposition is superior as one can assume that the deep parts of the layer have experienced the same annealing conditions as the top parts. This also implies more uniform layer morphology changes than post-deposition annealing, where heat has to be transported to the deeper parts of the layer. The resulting layer thickness is highly dependent on precursor concentration and spray time, while being less influenced by the substrate-nozzle distance and/or dispersion oxygen flow. Precursor concentration has an upper limit determined by solubility of the metal and, therefore, can be impracticable to tune the layer thickness. Other precursors such as Indium and Tungsten are limited by the solubility to a concentration of 0.1 M. The layer thickness is easily tuned by adapting the spray time. After approximately 1 min, the layer growth rate becomes constant for SnO_2_ and thus, layer thickness is predicted easily. While it is observed that the layer thickness is higher for shorter distance, this parameter needs careful tuning. We have attributed the effects of variation in distance to flame parameters’ particle concentration and temperature within the spray cone of FSP. The final conclusion is that dispersion oxygen has an influence on primary particle diameter rather than layer height. The tuning of dispersion oxygen could therefore be used to tune layer properties independent of thickness. It is possible to fabricate nanoparticle layers with direct deposition onto sensor substrates and tunable primary particle size.

### 3.4. Thermophoretic Deposition

Studies from recent years show that thermophoretic deposition is still an active field of scientific interest. Usually particles are assumed to be spherical for simplification. However, calculations of thermophoretic force on nonspherical particles do exist [47]. Although the difference in deposition rates between spheres and nonspherical particles with random orientation has been considered insignificant [48], a surprising effect is the reversal of thermophoretic force, then acting from cold to hot environment which has recently been demonstrated experimentally and numerically [49]. Though the test particle was in the range of cm, the Knudsen Number was kept low by lowering the pressure in the measurement chamber. For spheres of high thermal conductivity, a reversal of thermophoretic force is possible when the Knudsen Number is low. The simple approach to thermophoretic deposition is to neglect any contribution other than thermophoresis, though research about additional movement, i.e., by convection, does exist [50].

## 4. Conclusions

The thermophoretically fabricated SnO_2_ layers were investigated using SEM and FIB. The imaging techniques were utilized for extracting layer thickness of the particle when different flame parameters were considered (spray time vs. thickness, concentration vs. thickness, dispersed oxygen gas vs. thickness and substrate-nozzle distance vs. thickness, see Figure 6). It has been demonstrated that by choosing a certain set of parameters layer thickness (respectively) growth rate can be influenced. Additionally, the model of Tricoli and Elmøe has been proven to be an adequate tool for predicting the deposited mass of a layer. To acquire a certain thickness, it is best to vary spray time to avoid any undesired changes in primary particle size or porosity. For specific application of the deposited layer, i.e., Gas Sensing where high porosity is beneficial, the response time towards specific target gases can be optimized by tuning the layer thickness and primary particle size while maintaining the previously chosen growth rate of the sensing layer. However, our method of investigation has the drawback of breaking the layers, which makes it impractical to determine the thickness and porosity of the real layer used in functional experiments. Nondestructive methods should be considered for determination of layer thickness for functional layers to be used in further experiments.

Studies suggested that annealing of sensing layers is capable of improving stability and baseline resistance of gas sensing films while maintaining the benefits of nanocrystalline sensitive layers. Prior to annealing, the knowledge of film thickness and possible influences is still important as tuning the optimal parameters will be critical to optimize the thickness with regards to sensor response and response/recovery time. Future work is required to explore whether best sensing performance is reached with direct thermophoretic layer deposition, lamination or in-situ annealing.

## Figures and Tables

**Table 1:** Overview of gas phase thermophoretic deposition experiments conducted with constant and varying parameters. Last two columns show specific surface areas, specific particle sizes and crystallite sizes of the powders collected during deposition.

	Description	Precursor Conc. (M)	Nozzle to Substrate Distance (HAB) (mm)	Spray Time (s)	Disps. O_2_ (L/min)	Mean Layer Thickness (µm)	SSA, *d*_BET_ (m^2^/g), (nm)	XRD, *d*_XRD_ * (nm)
1	Sn-Precursor Concentration Dependent Functional Nanoparticle Layer	0.1	250	1200	5	23.2	143.9, 6.0 **	3.6 **
		0.5				113.2	99.4, 8.68	8.9
		1.0				165.4	71.8, 11.9	11.0
2	Nozzle-Substrate Distance Dependent Functional Nanoparticle Layer.	0.5	250	1200	5	113.2	99.4, 8.68	8.9
			220			116.3	83.2, 10.4	8.7
			200			131.9	88.8, 9.7	8.5
3	Time Dependent Functional Nanoparticle Layer	0.5	250	30	5	8.5	84.7, 10.2	7.8
				60		16.8		
				300		44.9		
				600		74.3		
				1200		152.1	91.7, 9.4	8.5
4	Dispersion O_2_ Dependent Functional Particle Layer	0.5	250	1200	3	116.7	66.5, 13.0	11.9
					4	124.2	68.6, 12.6	9.9
					5	108.8	99.4, 8.68	9.0
					6	99.2	96.1, 8.9	7.8
					7	112.3	104.8, 8.4	6.3

* The SnO_2_ crystallizes in tetragonal crystal system with a = b ≠ c, where a = b = 4.73 Å. The changes in the lattice parameter c (3.18 Å) were within the deviation of 3%. ** data taken from reference [32].

## Data Availability

Data is contained within the article or supplementary material. The data presented in this study are available in Supplementary Materials.

## Supplementary Materials

The following are available online at https://www.mdpi.com/article/10.3390/ma14092395/s1, Figure S1. XRD patterns of particles collected with varying precursor concentration. (a) particles obtained with a pre-cursor concentration of 0.1 and 0.5 M collected together and (b) particles obtained with a precursor concentration of 1.0 M, Figure S2. XRD patterns of particles collected with varying dispersion oxygen flow. (a) 3 L/min, (b) 4 L/min, (c) 5 L/min, (d) 6 L/min and (e) 7 L/min, Figure S3. XRD Patterns of powder collected at varying distance. (a) substrates during spray placed at 250 mm, (b) 220 mm and (c) 200 mm, Figure S4. XRD patterns of powder collected at various spray times. (a) powder of spray times 30, 60, 300 and 600 s collected together and (b) powder of 1200 s.
