# Supplementary material for: Control of Porous Layer Thickness in Thermophoretic Deposition of Nanoparticles
Source: Materials (Basel). 2021 May 4;14(9):2395. doi: 10.3390/ma14092395 (PMC8124515; doi:10.3390/ma14092395)
Supplement: Supplementary file 1 [file materials-14-02395-s001.zip › materials-1165100-supplementary.pdf]

Article

Control of Porous Layer Thickness in Thermophoretic Deposition of Nanoparticles

Malte Schalk [1,2], Suman Pokhrel [1,2], Marco Schowalter [3], Andreas Rosenauer [3] and Lutz Mädler [1,2,*]

[1] Faculty of Production Engineering, University of Bremen, 28359 Bremen, Germany; m.schalk@iwt.uni-bremen.de (M.S.); spokhrel@iwt.uni-bremen.de (S.P.)
[2] Leibniz Institute for Materials Engineering IWT, 28359 Bremen, Germany
[3] Institute of Solid State Physics, University of Bremen, 28359 Bremen, Germany; schowalter@ifp.uni-bremen.de (M.S.); rosenauer@ifp.uni-bremen.de (A.R.)
* Correspondence: lmaedler@iwt.uni-bremen.de; Tel.: +49-42121851200

Abstract: The film thickness plays an important role in the performance of materials applicable to different technologies including chemical sensors, catalysis and/or energy materials. The relationship between the surface and volume of the functional layers is key to high performance evaluations. Here we demonstrate the thermophoretic deposition of different thicknesses of the functional layers designed using flame combustion of tin 2-ethylhexanoate dissolved in xylene, and measurement of thickness by scanning electron microscopy and focused ion beam. The parameters such as spray fluid concentration (differing Sn^{2+} content), substrate-nozzle distance and time of the spray were considered to investigate the layer growth. The results showed ~ 23, 124 and 161 μm thickness of the SnO_2 layer after flame spray of 0.1, 0.5 M and 1.0 M tin 2-EHA-Xylene solutions for 1200 s. While Sn^{2+} concentration was 0.5 M for all the flame sprays, the substrates placed at 250, 220 and 200 mm from the flame nozzle had layer thicknesses of 113, 116 and 132 μm, respectively. Spray time dependent thickness growth showed a linear increase from 8.5 to 152.1 μm when the substrates were flame sprayed for 30 s to 1200 s using 0.5 M tin 2-EHA-Xylene solutions. Changing the dispersion oxygen flow (3–7 L/min) had almost no effect on layer thickness. Layers fabricated were compared to a model found in literature, which seems to describe the thickness well in the domain of varied parameters. It turned out that primary particle size deposited on the substrate can be tuned without altering the layer thickness and with little effect on porosity. Applications depending on porosity, such as catalysis or gas sensing, can benefit from tuning the layer thickness and primary particle size.

Keywords: flame spray pyrolysis; nanoparticles; thermophoretic deposition; film thickness

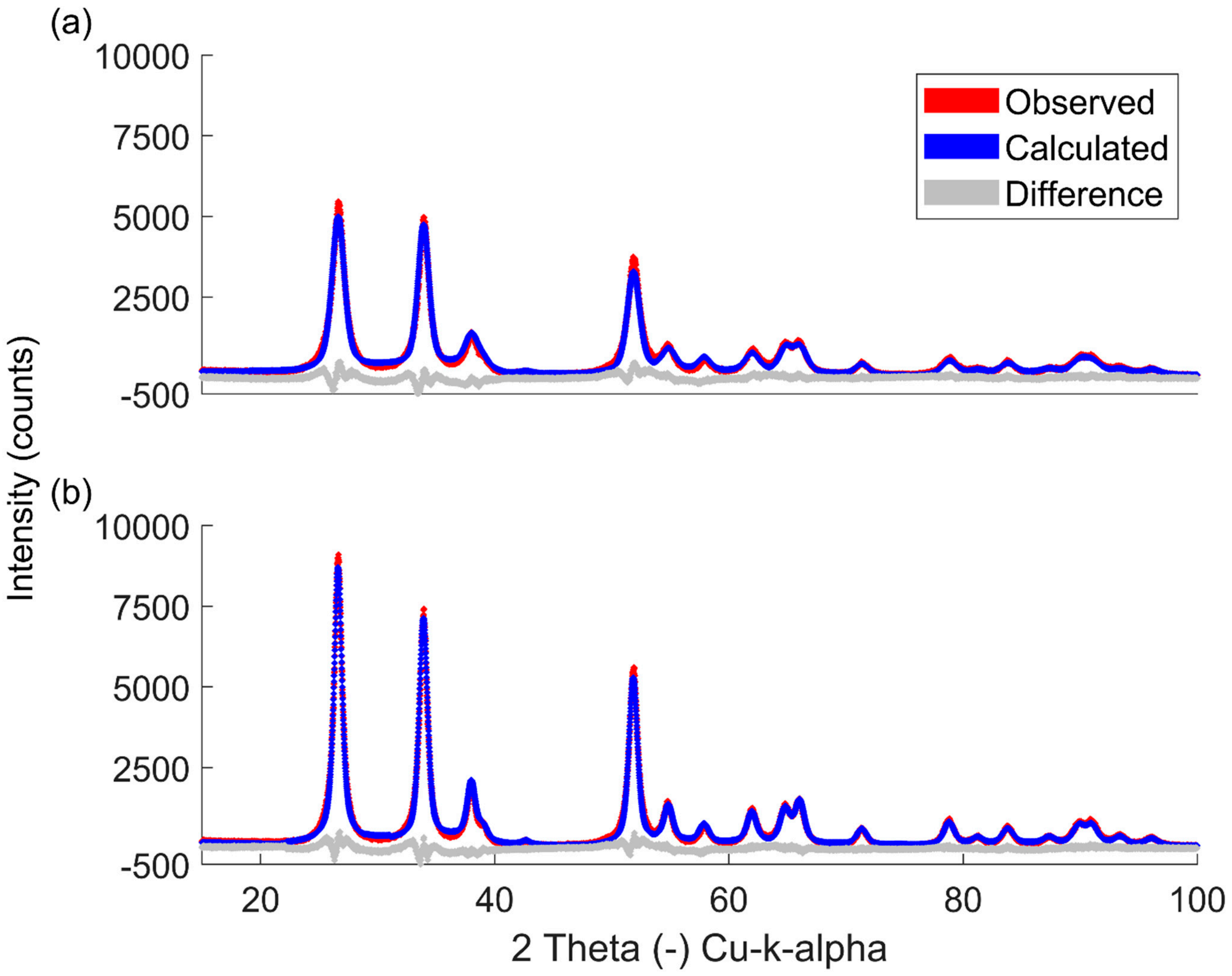

Fig. S1: *XRD patterns of particles collected with varying precursor concentration. (a) particles obtained with a precursor concentration of 0.1 and 0.5 M collected together and (b) particles obtained with a precursor concentration of 1.0 M*

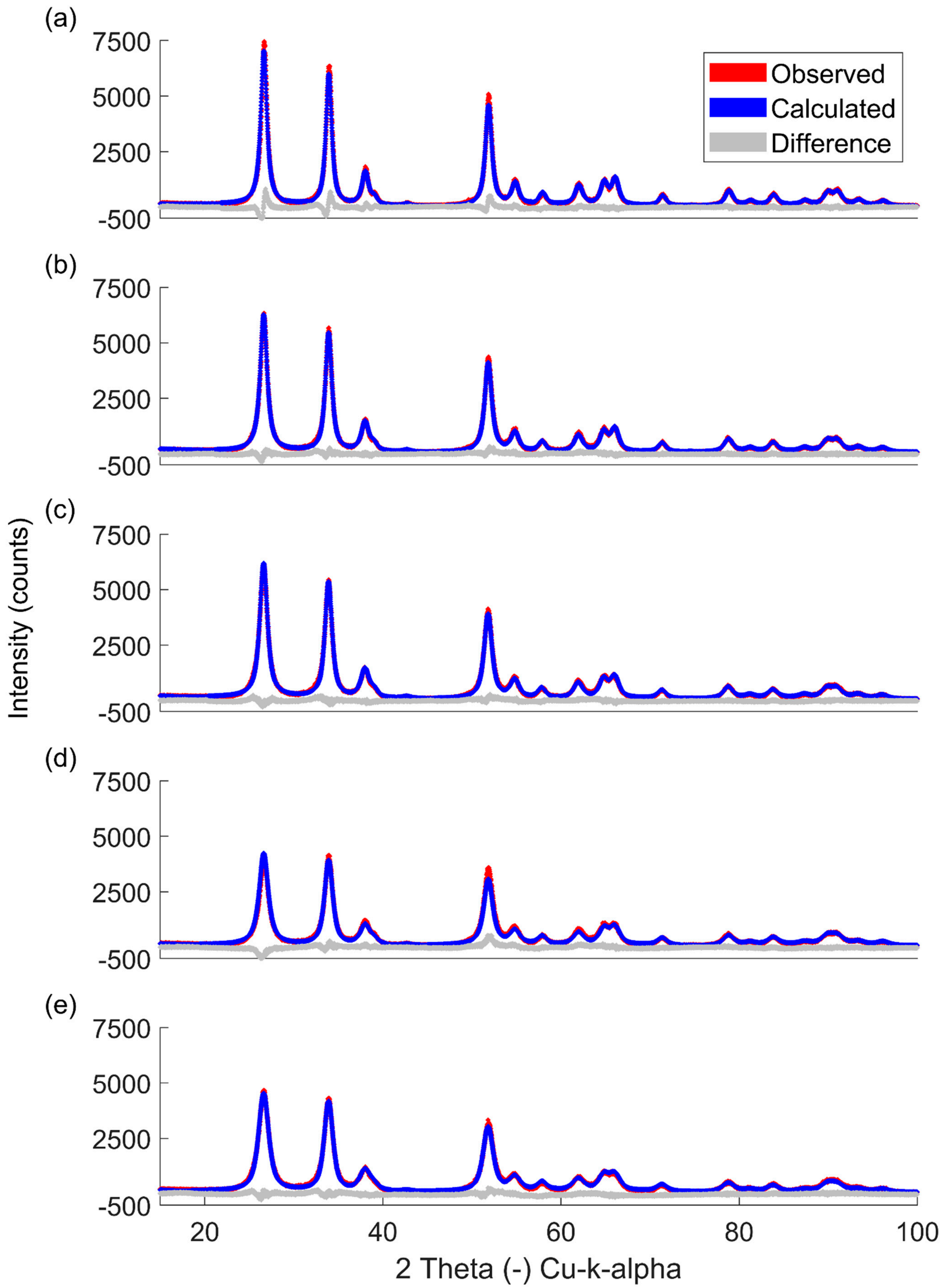
(a)
(b)
(c)
(d)
(e)
7500
5000
2500
-500
Observed
Calculated
Difference
Intensity (counts)
20
40
60
80
100
2 Theta (-) Cu-k-alpha

Fig. S2: *XRD patterns of particles collected with varying dispersion oxygen flow. (a) 3 l/min, (b) 4 l/min, (c) 5 l/min, (d) 6 l/min and (e) 7 l/min*

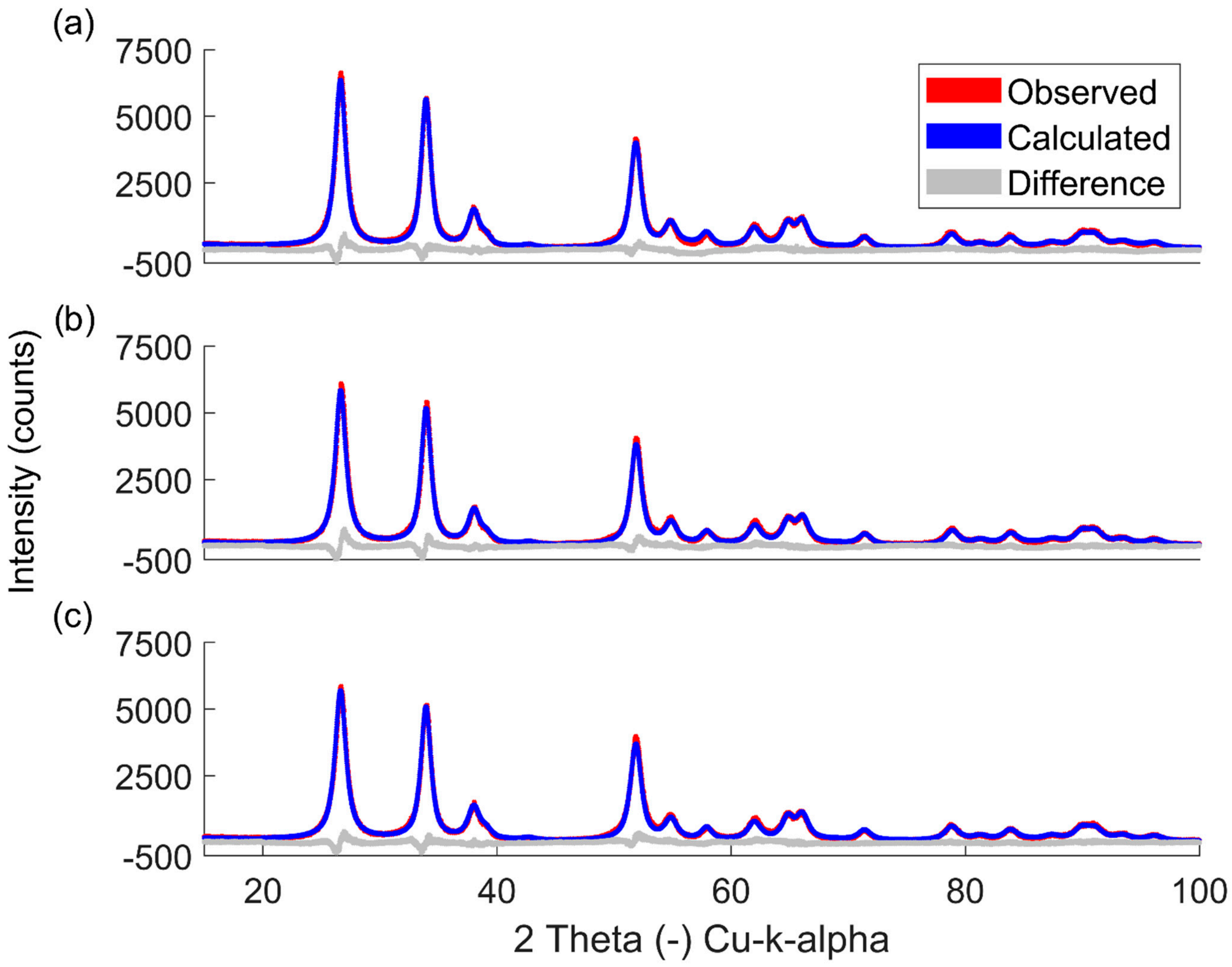

Fig. S3: *XRD Patterns of powder collected at varying distance. (a) substrates during spray placed at 250 mm, (b) 220 mm and (c) 200 mm*

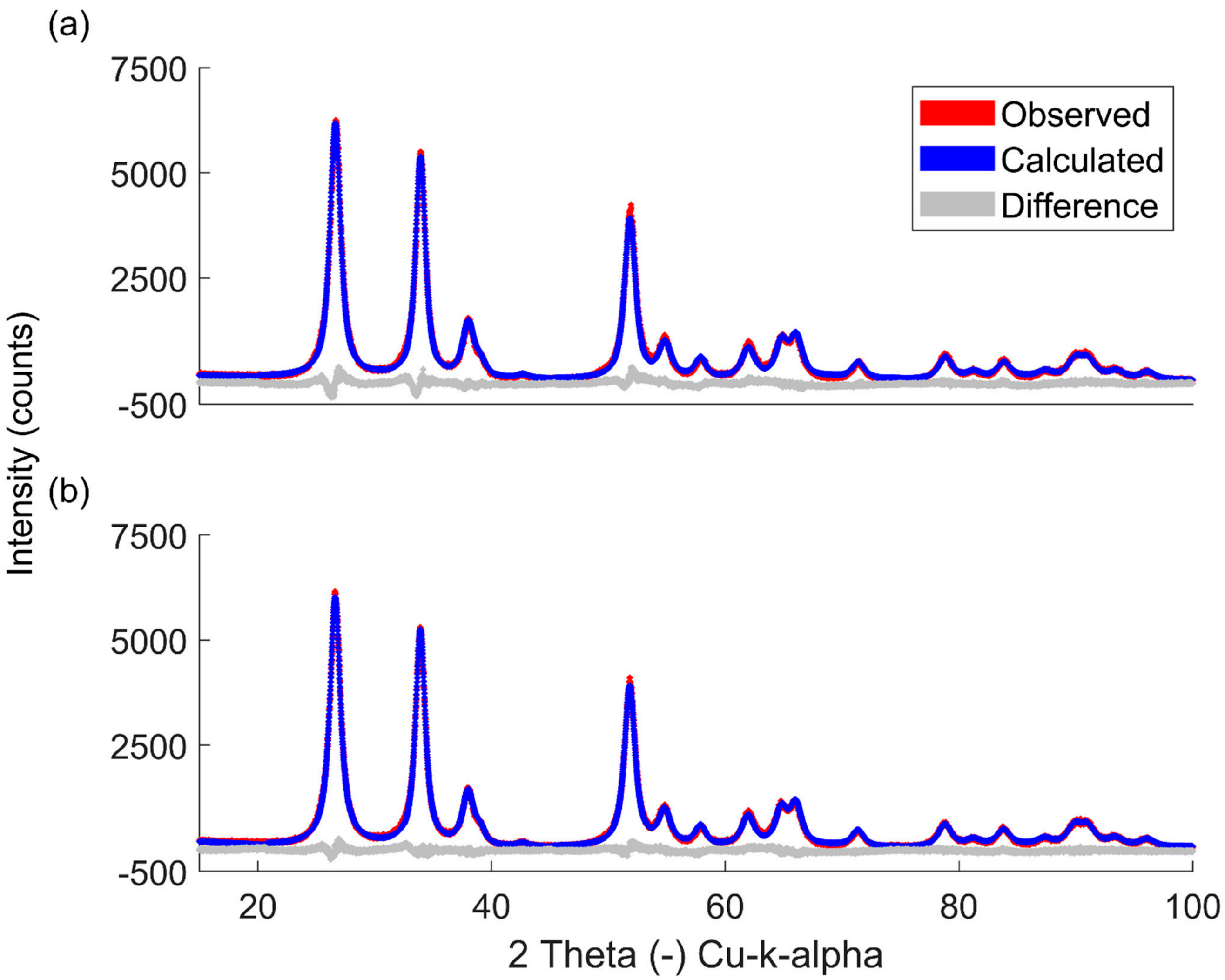

Fig. S4: *XRD patterns of powder collected at various spray times. (a) powder of spray times 30, 60, 300 and 600 s collected together and (b) powder of 1200 s.*
